# NOX4-mediated astrocyte ferroptosis in Alzheimer’s disease

**DOI:** 10.1186/s13578-024-01266-w

**Published:** 2024-07-02

**Authors:** Yasenjiang Maimaiti, Ting Su, Zhanying Zhang, Lingling Ma, Yuan Zhang, Hong Xu

**Affiliations:** Gerontology Center, People’s Hospital of Xinjiang Uygur Autonomous Region, No.91 Tianchi Road, Urumqi, Xinjiang China

**Keywords:** Single-cell sequencing, Ferroptosis, NADPH oxidase 4, Astrocytes, Alzheimer’s disease, Differential gene analysis, Immunofluorescence staining, Mouse model validation

## Abstract

This study investigates NADPH oxidase 4 (NOX4) involvement in iron-mediated astrocyte cell death in Alzheimer’s Disease (AD) using single-cell sequencing data and transcriptomes. We analyzed AD single-cell RNA sequencing data, identified astrocyte marker genes, and explored biological processes in astrocytes. We integrated AD-related chip data with ferroptosis-related genes, highlighting NOX4. We validated NOX4’s role in ferroptosis and AD in vitro and in vivo. Astrocyte marker genes were enriched in AD, emphasizing their role. NOX4 emerged as a crucial player in astrocytic ferroptosis in AD. Silencing NOX4 mitigated ferroptosis, improved cognition, reduced Aβ and p-Tau levels, and alleviated mitochondrial abnormalities. NOX4 promotes astrocytic ferroptosis, underscoring its significance in AD progression.

**Supplementary Information:**

The online version contains supplementary material available at 10.1186/s13578-024-01266-w.

## Introduction

Alzheimer’s disease (AD) is a prevalent neurodegenerative condition characterized by memory loss, cognitive decline, and impaired behavioral abilities [1–3]. Despite some advances in understanding AD’s pathological mechanisms, the specific molecular underpinnings remain elusive, and effective treatments targeting its root causes are lacking [1, 4–6]. A comprehensive investigation into AD’s pathogenesis holds paramount importance for its prevention and treatment. In recent years, ferroptosis, an emerging form of cell death reliant on iron, has garnered significant attention in the realm of neurodegenerative diseases [7–10]. Astrocytes are one of the most abundant subtypes of glial cells in the central nervous system [11, 12]. They are associated with brain development and function, such as regulating synaptic formation and function, controlling neurotransmitter release and uptake, producing trophic factors, and maintaining neuronal survival [13–16]. As major glial cells in the central nervous system, astrocytes also play a role in various physiological and pathological processes in the brain and may have significant implications for the pathogenesis of AD [17–19]. Previous studies have indicated higher levels of astrocyte damage in Alzheimer’s disease patients [20].

Ferroptosis, characterized by heightened lipid peroxidation and iron-dependent reactive oxygen species generation, constitutes a novel iron-dependent cell demise pathway [21–23]. There is a close relationship between oxidative stress and iron death. Both iron excess and deficiency can induce oxidative stress, leading to cell death and other related diseases [24]. Studies suggest that iron death may play a significant role in neurodegenerative diseases, including Alzheimer’s disease [25]. Furthermore, literature indicates the presence of high concentrations of iron in the brains of AD patients and transgenic mouse models, where excess iron can exacerbate oxidative damage and cause cognitive impairment. Disruption of iron homeostasis is considered to be associated with Alzheimer’s disease [26, 27]. Increasing evidence indicates that iron death can lead to AD-mediated neuronal cell death [28]. Nevertheless, iron’s precise roles and regulatory mechanisms in Alzheimer’s, particularly its interplay with astrocytes, remain enigmatic [29]. Star-shaped glial cells play pivotal roles in the central nervous system’s physiological and pathological processes, with certain key regulatory genes or pathways potentially offering valuable insights into Alzheimer’s pathogenesis [30–32].

Single-cell sequencing technology, noted for its remarkable sensitivity and high resolution, has gained widespread adoption in biomedical research [33–35]. This innovative approach facilitates the analysis of gene expression and regulatory networks at the single-cell level, revealing cellular heterogeneity and dynamic changes under physiological and pathological conditions [36]. Single-cell sequencing affords the capacity to dissect distinct neural cell types, including neurons and glial cells, and their roles in disease progression, offering invaluable insights into neurodegenerative conditions such as Alzheimer’s [37].

NADPH oxidase 4 (NOX4), an enzyme with protein catalytic activity that generates reactive oxygen species (ROS), exerts pivotal roles in various physiological and pathological processes encompassing cell proliferation, migration, and cell death [38–40]. Current research highlights NOX4’s potential significance in neurodegenerative diseases, including Parkinson’s and Alzheimer’s diseases. Nonetheless, the precise mechanisms by which NOX4 influences Alzheimer’s, particularly its association with astrocytic iron-mediated cell death, remain to be fully elucidated [41, 42].

This study’s primary objective is to elucidate the critical role of NADPH oxidase 4 (NOX4) in iron-triggered astrocytic cell death and its implications for Alzheimer’s disease (AD) pathogenesis. Employing single-cell sequencing technology, the GEO database, and transcriptome sequencing data, this study delves deeply into the cellular populations and associated genes of AD patients. Researchers meticulously label and analyze various cell types, identifying distinctive marker genes for astrocytes. Furthermore, this study uncovers NOX4’s pivotal role in iron-induced astrocyte demise. These findings substantially augment our comprehension of Alzheimer’s disease etiology, particularly elucidating the nexus between NOX4, astrocytic iron-mediated death, and AD. Importantly, these insights hold clinical relevance for the diagnosis and treatment of Alzheimer’s disease.

## Materials and methods

### Transcriptome sequencing data acquisition

The single-cell transcriptome sequencing data of AD-related samples in the GSE164089 dataset was analyzed using the Seurat package in R software. To ensure data quality, quality control criteria were applied, including nFeature_RNA > 500, 1000 < nCount_RNA < 20,000, and percent.mt < 10%. Additionally, the top 1000 highly variable genes were selected based on their variance. Furthermore, AD-related microarray dataset GSE48350 was obtained from the GEO database (https://www.ncbi.nlm.nih.gov/geo/). This dataset consists of 173 normal brain tissue samples and 80 AD brain tissue samples [43].

### TSNE clustering analysis

To reduce the dimensionality of scRNA-Seq datasets, we employ principal component analysis (PCA) based on the top 1000 genes with the highest variance in expression. We used the Elbowplot function of the Seurat package and selected the top 15 principal components for downstream analysis. Using the FindClusters function provided by Seurat, we identified different subpopulations of cells with the default resolution (res = 0.5). Next, we use the t-SNE algorithm to reduce nonlinear dimensionality on scRNA-seq sequencing data. We also used the Seurat package to identify marker genes for individual cell subpopulations and combined the single package with the online website CellMarker (http://xteam.xbio.top/CellMarker) for cell type annotation analysis [44, 45].

### GO and KEGG enrichment analysis

The differential expression genes (DEGs) were subjected to Gene Ontology (GO) and Kyoto Encyclopedia of Genes and Genomes (KEGG) enrichment analysis using the “clusterProfiler”, “org.Hs.eg.db”, “enrichplot”, and “ggplot2” packages in the R language. Bubble plots and circular plots were generated to visualize the enrichment results of the three categories, namely Biological Processes (BP), Cellular Components (CC), and Molecular Functions (MF), in the Gene Ontology (GO). Additionally, a bubble plot was generated to display the enrichment results of the KEGG pathway analysis [46].

### Differential gene expression screening

The “limma” package in R software was utilized for the selection of differentially expressed genes. Differentially expressed genes between normal samples and AD samples were filtered based on the criteria |logFC| > 0 and P.adjust < 0.05 [47].

### Lentivirus infection

To construct a lentivirus-mediated NOX4 silencing vector, the pSIH1-H1-copGFP (sh-) interference vector (catalog number SI501A-1, System Biosciences, USA) was purchased. The silencing sequence can be found in Table S1. The lentiviral particles carrying the vector were packaged into HEK-293T cells (CRL-3216, ATCC, USA) using the lentivirus packaging reagent kit (catalog number A35684CN, Invitrogen, USA). After 48 h, the supernatant was collected to obtain lentivirus with a titer of 1 × 10^8^ TU/ml. Researchers interested in rapidly and efficiently constructing a lentiviral vector that mediates NOX4 silencing may consider adopting the methodology utilized in our laboratory [48, 49].

### Cell culture and screening

Human normal astrocytes were purchased from ATCC (ATCC, USA) and cultured in human astrocyte medium (catalog number 1801, ScienCell, USA). The medium consisted of a basal medium (catalog number 1801), 2% (v/v) fetal bovine serum (FBS, catalog number 0010), 1% (v/v) astrocyte growth supplement (AGS, catalog number 1852), and 1% (v/v) penicillin/streptomycin solution (P/S, catalog number 0503). The cells were cultured at 37 °C in a 5% CO2 incubator. To simulate Aβ-induced neuronal injury, the cells were treated with Aβ25–35 peptide (catalog number A107853-25 mg, Aladdin, Shanghai, China) at a concentration of 20 μM for 24 h. The cells were divided into the following groups: control, AD, AD + sh-NC (infected with negative control lentivirus expressing sh-NC), and AD + sh-NOX4 (infected with lentivirus expressing sh-NOX4). After adding 1 × 10^5^ TU lentivirus to the astrocytes, the cells were incubated for 48 h, except for the control group, which was incubated for an additional 24 h in the medium containing 20 μM Aβ25–35 peptide [50, 51].

### Alzheimer’s disease APP/PS1 mouse model

The male transgenic mice with overexpression of human amyloid precursor protein (APP) and mutant forms of presenilin 1 (PS1) were purchased from Jackson Laboratory (Bar Harbor, ME, USA, Stock #034829). The wild-type C57 male mice were purchased from Weitonlihua Experimental Animal Technology Co., Ltd. in Beijing, China for the Alzheimer’s disease model experiments. They were maintained under non-pathogenic conditions at a temperature of 26–28 ℃ and humidity of 50–65%, with free access to food and water. All mice were acclimated for one week prior to the experiments. The experimental procedures were conducted in accordance with ethical standards and were approved by our institution’s Animal Ethics Committee.

For the experiments, the mice were randomly divided into five groups: WT, APP/PS1, APP/PS1 + sh-NC, APP/PS1 + sh-NOX4, and APP/PS1 + sh-NOX4 + erastin, with six mice in each group. To silence NOX4 in the neurons in vivo, sh-NOX4 (4 × 10^5^ TU) or sh-NC (4 × 10^5^ TU) was slowly injected into the bilateral hippocampi of APP/PS1 mice. In the APP/PS1 + sh-NOX4 + erastin group, erastin (10 μM; HY-15,763, MedChem Express, New Jersey, USA) was dissolved in a water bath at 37℃ with gentle shaking, and then 5% dimethyl sulfoxide with corn oil (C8267, Sigma-Aldrich, USA) was added. The mice were treated for 20 days. At the end of the experiment, all mice were euthanized with an overdose of anesthetic (pentobarbital sodium). The tissues were processed by perfusing the ascending aorta with a 0.9% sodium chloride solution, followed by fixation of the brain tissues in 4% paraformaldehyde solution and embedding in paraffin [38, 52–54]. The behavior test experiment was finished, and the above processing methods were executed.

### Immunofluorescent staining

The brain tissue embedded in paraffin was sectioned into 4 μm thick slices and permeabilized using 0.5% Triton-X (T8787, Sigma-Aldrich, USA). The slices were then blocked in CAS-Block™ tissue blocking reagent (008120, Thermo Fisher Scientific, Waltham, MA, USA). Immunostaining was performed using the following antibodies: rabbit anti-GFAP antibody (ab207165, Abcam, Cambridge, UK), rabbit anti-NOX4 antibody (MA5-32090, 1:50, ThermoFisher, USA), rabbit anti-4-HNE antibody (MA5-45789, 1:50, ThermoFisher, USA), and rabbit anti-malondialdehyde (MDA) antibody (MA5-45803, 1:50, ThermoFisher, USA), as well as rabbit monoclonal anti-GFAP antibody (ab207165, Abcam, Cambridge, UK). The slices were then incubated with secondary antibodies goat anti-rabbit IgG (H + L) Alexa Fluor 488 (A11008, 1:100, Thermo Fisher Scientific, USA) and goat anti-mouse IgG (H&L) Texas Red (ab6787, 1:100, Abcam, Cambridge, UK) at 25 °C for 2 h. Nuclear staining was performed using Fluoroshield™ with DAPI (F6057, Sigma-Aldrich, USA). The stained brain tissue sections were analyzed using THUNDER Imager Tissue (Leica Microsystems Ltd., Wetzlar, Germany) and quantified using LAS X imaging software (Leica Microsystems Ltd, Wetzlar, Germany) and ImageJ software v1.52a (Bethesda, MD, USA) [38].

### Flow cytometric cell sorting

The mouse brain tissue samples were cut into small pieces and digested at 37 °C in PBS solution containing 0.8 mg/mL Collagenase IV (Merck, C4-BIOC, USA). After a wash with PBS buffer, the cell suspension was filtered through a 50 μm sieve to remove residual solid components such as organic matter and neurons. The cell suspension was then centrifuged at 1000 rpm for 5 min. The supernatant was discarded, and the cell pellet was retained. Washing with cell culture medium was performed, followed by grinding the remaining tissue cell clusters using a homogenizer. After repeated washes, the cell suspension was added to approximately 1 mL of washing buffer for selection. Subsequently, the cell suspension was placed in a column containing magnetic beads labeled with S100β antibody.

In the spatial sliding column, cells were bound to the S100β antibody (# 9550 S, Cell Signaling Technology, Danvers, MA, USA). Through negative selection, non-stellar glial cells were removed while stellar glial cells were retained. Subsequent washing steps were performed to eliminate cells and impurities not bound to the magnetic beads. The cell suspension was then transferred to a sterile culture dish for inspecting the integrity and activity of stellar glial cells under a microscope. After identifying and collecting the target cell samples, the cells were fixed using PBS solution containing 3.7% formaldehyde. Permeabilization of the fixed cells was done using 0.1% Triton X-100, followed by labeling of the stellar glial cells using GFAP antibody (ab207165, Abcam, Cambridge, UK). The labeled cell samples were injected into a flow cytometer, and cell fluorescence intensity was measured by laser excitation to obtain a purity of 90% for stellar glial cells. Finally, the collected stellar glial cells were subjected to Western blot, lipid peroxidation detection, and measurement of iron ion content using the same culture conditions [55].

### Western blot

Tissue total protein was extracted using RIPA lysis buffer (P0013C, Beyotime, Shanghai, China) containing PMSF. The extraction process involved incubation on ice for 30 min, followed by centrifugation at 4 °C and 8000 g for 10 min to collect the supernatant. The total protein concentration was measured using a BCA assay kit (Catalog number: 23,227, ThermoFisher, USA). 50 μg of protein was dissolved in 2x SDS loading buffer and boiled for 5 min at 100 °C prior to SDS-PAGE gel electrophoresis. The proteins were then transferred to a PVDF membrane.

The PVDF membrane was blocked with 5% non-fat milk at room temperature for 1 h, followed by incubation overnight at 4 °C with the diluted primary antibodies: rabbit anti-GPX4 (ab125066, 1:1000, Abcam, Cambridge, UK), rabbit anti-NOX4 (MA5-32090, 1:1000, ThermoFisher, USA), rabbit anti-4-HNE antibody (MA5-45789, 1:1000, ThermoFisher, USA), rabbit anti-malondialdehyde (MDA) antibody (MA5-45803, 1:1000, ThermoFisher, USA), rabbit anti-SAT1 antibody (ab105220, 1:500, Abcam, Cambridge, UK), rabbit anti-FTH1 antibody (PA5-27500, 1:500, ThermoFisher, USA), rabbit anti-SLC7A11 (711,589, 1:1000, ThermoFisher, USA), rabbit anti-ACSL4 (PA5-27137, 1:1000, ThermoFisher, USA), and rabbit anti-GAPDH (ab181602, 1:10000, Abcam, Cambridge, UK) as internal references. Subsequently, the membrane was washed three times with TBST for 10 min each, incubated for 1 h with an HRP-conjugated goat anti-rabbit IgG H&L secondary antibody (ab97051, 1:2000, Abcam, Cambridge, UK), and then placed on a clean glass slide after TBST rinsing.

ECL fluorescence detection reagents from the ECL assay kit (Catalog number: abs920, Aibikeshin (Shanghai) Biotechnology Co., Ltd., Shanghai, China) were mixed in equal amounts of A and B solution. The mixture was then added onto the membrane and imaged using the Bio-Rad imaging system (Bio-Rad, USA) in a darkroom. Finally, the Quantity One v4.6.2 software was used for analysis, with the grayscale value of the corresponding protein bands normalized to the grayscale value of the GAPDH protein band to represent the relative protein content [56]. Repeat each experiment three times and take the average value.

### Determination of GSH content

The GSH content in human and murine astrocytes was determined using the GSH assay kit (A006-2-1, Nanjing Institute of Biotechnology, China) according to the manufacturer’s instructions. Initially, the collected cells were washed 1–2 times with PBS and pelleted by low-speed centrifugation. The pellet was then resuspended in PBS buffer. Subsequently, the cells were manually homogenized for detection after cell disruption [57].

### MDA determination

Ferroptosis is a form of cell death caused by the accumulation of lipid peroxidation products on the cell membrane. Therefore, the level of malondialdehyde (MDA), a lipid peroxidation product, can serve as an indicator of ferroptosis. In this study, we evaluated the MDA content in astrocytes using the MDA assay kit (A003-4-1, China) produced by the Nanjing Institute of Biotechnology. The assay was conducted following the manufacturer’s instructions, and absorbance at 530 nm was measured using a microplate reader [58, 59].

### Iron content determination

The iron ion content in astrocytes can be measured using the iron assay kit (E1042, Beijing Pulei Gene Technology Co., Ltd., China) as instructed by the manufacturer. The procedure is as follows: First, collect the samples and wash them with PBS, then lyse the cells. Next, add Solution A to the collected lysate, mix, and incubate at 60 °C for 1 h. Finally, add the iron ion detection reagent, mix, and incubate for 30 min. Transfer 200 μl of the solution into a 96-well plate, and measure the absorbance at a wavelength of 550 nm [60].

To examine the effect of the orange fluorescent iron probe FerroOrange on ferrous ions in human and murine astrocytes, we first co-incubated the cells with Hoechst 33,342 (#4082, Cell Signaling Technology, Danvers, MA, USA) for 15 min, followed by three washes with PBS. Then, after a 30-minute incubation with 1 μM FerroOrange (#36,104, Cell Signaling Technology, Danvers, MA, USA), we washed the cells three times with PBS and used a laser confocal microscope (Olympus, Tokyo, Japan) to capture cell images. Fluorescence signals were observed and analyzed by exciting at 561 nm and detecting emission light between 570 and 620 nm, represented as the color orange [61].

### GPX4 activity assay

According to the manufacturer’s instructions, the GPX4 ELISA kit was utilized to measure the activity of GPX4. For the cell sample (ml060706, mlbio, China), centrifugation at 1000×g for 10 min was performed to remove particles and aggregates, thereby obtaining the cell supernatant. Subsequently, the sample of interest and biotin-labeled antibody were co-incubated, followed by washing and addition of the avidin-labeled HRP. Afterwards, unbound enzyme complex was removed through incubation and washing, and substrate A, B, and the enzyme complex were concurrently added to generate a color reaction. The intensity of color corresponds to the concentration of the target substance in the sample. In the case of mouse serum sample (ml057982, mlbio, Shanghai), blood was collected in a tube without pyrogens and endotoxins. Centrifugation at 1000×g for 10 min was utilized to carefully and rapidly separate the serum from the red blood cells. Similar to the cell sample procedure, the sample of interest and biotin-labeled antibody were co-incubated, followed by washing and addition of the avidin-labeled HRP. Unbound enzyme complex was subsequently removed through incubation and washing, and substrate A, B, and the enzyme complex were concurrently added to generate a color reaction. The intensity of color corresponds to the concentration of the target substance in the sample [60].

### Lipid peroxidation detection

For the detection of lipid peroxidation during ferroptosis, mouse astrocytes were first collected by centrifugation. Subsequently, they were washed twice with PBS for 5 min each, and then 1 mL of BODIPY 581/591 C11 working solution was added, followed by incubation at room temperature for 15 min. The mixture was then centrifuged at 400 g for 3–4 min at 4 ℃, and the supernatant was discarded. The cells were washed again with PBS for 5 min, repeated twice. After resuspending the cells in 1 mL of serum-free culture medium, they were analyzed using a flow cytometer. During flow cytometry analysis, signals corresponding to 505–550 nm were measured in the FL1 channel, and signals above 580 nm were measured in the FL2 channel. When BODIPY 581/591 C11 undergoes an oxidation-reduction reaction with intracellular ROS, its maximum fluorescence emission shifts from around 590 nm to approximately 510 nm, and it is proportional to the generation of lipid reactive oxygen species (ROS) [62, 63].

### CCK-8

This study employed the CCK-8 assay kit (catalog number: CA1210, Beijing Solaibao Technology Co., Ltd., Beijing, China) for cell proliferation experiments. We first take cells in the logarithmic growth phase, with 1 × 104 cells seeded in each well and pre-cultured in a 96-well plate for 24 h. Afterward, the cells were transfected according to grouping. After transfection, 10μL of CCK-8 reagent was added at 48 h, respectively. Incubate for 3 h at 37 °C, then measure the absorbance values at 450 nm wavelength for each well on the spectrophotometer. The high or low absorbance values reflect the proliferation of cells in the culture medium. We create bar charts for each group to display the cell viability and present the experimental results [64].

### Morris water maze experiment

The Morris water maze test consisted of four platform trials and one probe trial conducted over five consecutive days. The movement trajectories of mice were recorded using video and analyzed using image analysis software (ANYMaze, Stoelting). At a temperature of 22–24℃, a circular pool filled with water containing titanium dioxide was used, with a platform positioned approximately 1 centimeter below the water surface in the first quadrant. In the platform trials, mice were placed in the water in one of the four quadrants. The time taken for a mouse to find and remain on the platform for 5 s after entering the water was recorded as the escape latency, along with the swimming path. If a mouse failed to find the platform within 60 s, the escape latency was recorded as 60 s. In the probe trial, the platform was removed, and the mice were allowed to freely swim in the pool for 60 s. We recorded the swimming paths, time spent in the target quadrant, swimming distance, and time spent in each quadrant [59].

### Immunohistochemistry staining

The brain tissue of each group of mice was embedded and sliced, followed by 20 min of baking at 60 °C. Subsequently, the slices were soaked in xylene for 15 min, with a change of xylene followed by another 15 min of soaking. After a 5-minute immersion in absolute alcohol, it was changed again for another 5 min. Then, the slices were hydrated in 95% and 70% ethanol for 10 min each. 3% H2O2 was added dropwise onto each slice to block endogenous peroxidase activity and incubated at room temperature for 10 min. Citrate buffer was then added, and the slices were microwaved for 3 min, followed by a 10-minute room temperature incubation with antigen retrieval solution and washed with PBS three times. Normal goat serum blocking solution (E510009, Shanghai Bioengineering Co., Ltd., China) was added and incubated at room temperature for 20 min, followed by overnight incubation at 4 °C with the following primary antibodies: mouse Aβ (ab230297, 1:200, Abcam, Cambridge, UK) and rabbit p-Tau (S396) (ab32057, 1:1000, Abcam, Cambridge, UK). After three washes with PBS, the slices were incubated with goat anti-rabbit IgG (ab6721, 1:1000, Abcam, UK) and goat anti-mouse IgG (ab150113, 1:500, Abcam, UK) secondary antibodies for 30 min and washed with PBS again. DAB chromogenic reagent kit (P0203, Beyotime, Shanghai, China) was used by adding a drop of each A, B, and C reagent onto the samples for 6 min of color development, followed by staining with hematoxylin for 30 s. Subsequently, the slices were dehydrated in 70%, 80%, 90%, 95% ethanol, and absolute ethanol for 2 min each. Finally, they were treated with xylene for 5 min, soaked twice, and then sealed with neutral resin. The slices were observed under an upright microscope (BX63, Olympus, Japan) [59].

### Congo red staining

Due to the deposition of Aβ plaques in the brain, Congo red staining was performed on brain tissue samples. The brain tissue was sliced from paraffin-embedded tissue blocks into sections with a thickness of 4 μm. Following the manufacturer’s instructions, the samples were stained with Congo red. Subsequently, the sections were observed and photographed at 400x and 100x magnification using the ECLIPSE Ci-L plus optical microscope from Nikon Co., Ltd., Japan, facilitating research and analysis [59].

### Transmission electron microscope (TEM)

To observe the microstructure of brain tissue using transmission electron microscopy, the following procedures were employed: Initially, brain tissue was perfused with 2.5% glutaraldehyde in 0.1 M sodium phosphate buffer (pH 7.4), followed by extraction and washing with phosphate buffer. Subsequently, small cubic brain tissue samples with a volume not exceeding 1 mm³ were fixed overnight at 4 °C. Afterward, the samples were sliced into sections (with a thickness of 50 nm) using an ultramicrotome and stained with uranyl acetate and lead citrate. Finally, the sections were observed using the JEM-1011 transmission electron microscope (JEOL Ltd., Tokyo, Japan) [65, 66].

### Statistical analysis

We used SPSS 22.0 statistical software (SPSS, Inc., Chicago, IL, USA) and GraphPad Prism 9.5 to analyze all the data. Data are presented as mean ± standard deviation (SD). A paired t-test should be used for comparing two groups, and a one-way analysis of variance (ANOVA) should be used to compare multiple groups. The homogeneity of variance test uses Levene’s method. Dunnett’s t-test and LSD-t-test are used for pairwise comparisons if the variances are homogeneous. Dunnett’s T3 test should be used if the variances are not homogeneous. *P* < 0.05 indicates that the difference between the two groups is statistically significant.

## Results

### Single-cell transcriptomic sequencing analysis revealed that astrocytes play a critical role in the pathogenesis and progression of Alzheimer’s disease

We analyzed a single-cell transcriptome sequencing dataset related to Alzheimer’s disease obtained from the GEO database, which included two AD samples (GSM4996461, GSM4996463) from GSE164089. Upon data integration using the Seurat package, the results revealed that the majority of cells had nFeature_RNA > 500, 1000 < nCount_RNA < 20,000, and percent.mt < 10% (Figure S1A). Following these criteria, we removed low-quality cells to obtain the expression matrix. Computational analysis of sequencing depth correlations indicated that the filtered cell data exhibited good quality (Figure S1B), permitting their utilization for subsequent analyses.

We analyzed the Alzheimer’s disease (AD)-related single-cell transcriptome sequencing dataset obtained from the GEO database, which includes two AD samples (GSM4996461, GSM4996463) from GSE164089. Upon data integration using the Seurat package, the results revealed that the majority of cells had nFeature_RNA > 500, 1000 < nCount_RNA < 20,000, and percent.mt < 10% (Figure S1A).

Following these criteria, we removed low-quality cells to obtain the expression matrix. Computational analysis of sequencing depth correlations indicated that the filtered cell data exhibited good quality (Figure S1B), permitting their utilization for subsequent analyses.

**Fig. 1:**
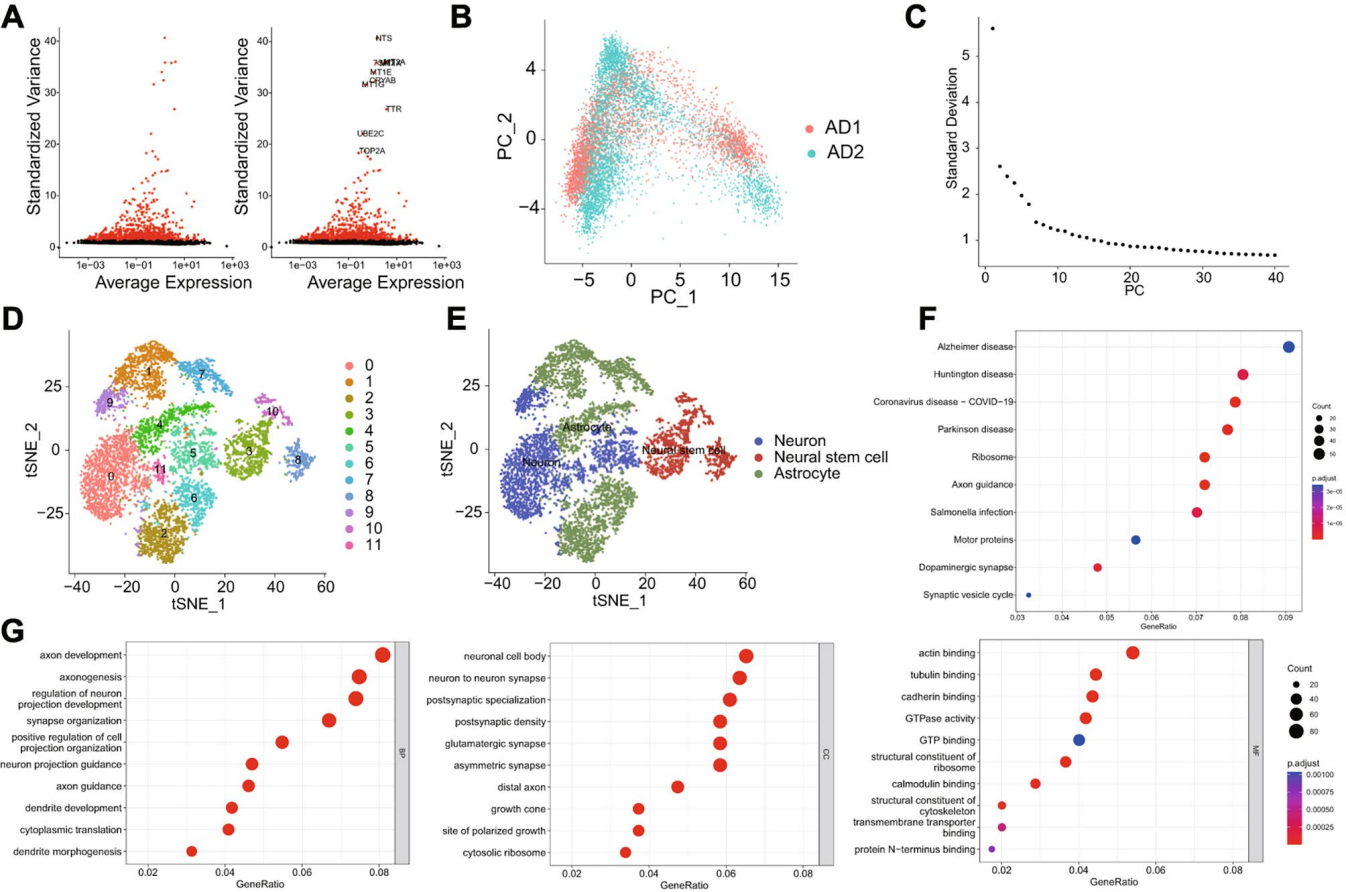
scRNA-seq Cell Clustering and Annotation Note: (**A**) Differential gene expression analysis identified highly variable genes, with red representing the top 1000 highly variable genes, black representing genes with low variability, and the top 10 gene names in highly variable genes. (**B**) Distribution of cells on PC_1 and PC_2, with each point representing a cell. (**C**) Distribution of standard deviation of PCs, with important PCs having more significant standard deviations. (**D**) Visual representation of tSNE clustering results showing the aggregation and distribution of cells from different sources, with each color representing a cluster. (**E**) Visualization of cell annotation results based on tSNE clustering, with each color representing a cell subpopulation. (**F**) KEGG pathway enrichment analysis of marker genes in astrocytes, with GeneRatio on the x-axis, KEGG functional terms on the y-axis, circle size indicating the number of enriched genes in the term, and color representing enrichment *p*-value. (**G**) GO functional analysis of marker genes in astrocytes at the biological process, cellular component, and molecular function levels, with GeneRatio on the x-axis, GO functional terms on the y-axis, circle size indicating the number of enriched genes in the term, and color representing enrichment *p*-value

We further analyze the filtered cells and identify highly variable genes based on gene expression variance. We selected the top 1000 genes with high variability in variance for downstream analysis (Fig. 1A). Afterward, we used principal component analysis (PCA) to reduce the dimensionality of the data linearly and presented the heatmap of the major correlated gene expression profiles of PC_1 to PC_6 (Figure S1C), as well as the distribution of cells in PC_1 and PC_2 (Fig. 1B). The results show that there is no noticeable batch effect among the samples. We then use ElbowPlot to sort the principal components (PCs) by the standard deviation (Fig. 1C). The results indicate that PC_1 - PC_15 could fully reflect the information in the selected highly variable genes and have good analytical significance.

In addition, we apply the t-SNE algorithm for nonlinear dimensionality reduction on the first 15 principal components. By clustering, we obtained 12 clusters (Fig. 1D) and extracted each cluster’s marker gene expression profiles (Figure S1D). Cell type annotation analysis was performed using the single package and the online website CellMarker (Fig. 1E) [44]. We have identified three types of cells, namely neurons, neural stem cells, and astrocytes. Clusters 3, 8, and 10 are annotated as neural stem cells, clusters 0, 5, 9, and 11 are annotated as neurons, and clusters 1, 2, 4, 6, and 7 are annotated as astrocytes.

Therefore, we performed KEGG and GO analysis on the marker genes of astrocytes. The KEGG analysis results showed (Fig. 1F): the marker genes of astrocytes were mainly enriched in entries related to Alzheimer’s disease, Huntington’s disease, Parkinson’s disease, and other diseases.

The results of the GO functional analysis (Fig. 1G) show that the glial cell marker genes are mainly enriched in biological processes (BP) such as axon development, axonogenesis, and regulation of neuronal projection development. Cellular components (CC) are mainly enriched in the neuronal cell body, synaptic junction, and postsynaptic specialization of neurons. In molecular function (MF), they are mainly enriched in actin binding, microtubule binding, and calcium binding, among others.

The above results indicate that astrocytes play a crucial role in the pathogenesis of Alzheimer’s disease.

### Transcriptomic sequencing analysis revealed that the NOX4 gene plays a critical role in the occurrence and progression of Alzheimer’s disease

**Fig. 2:**
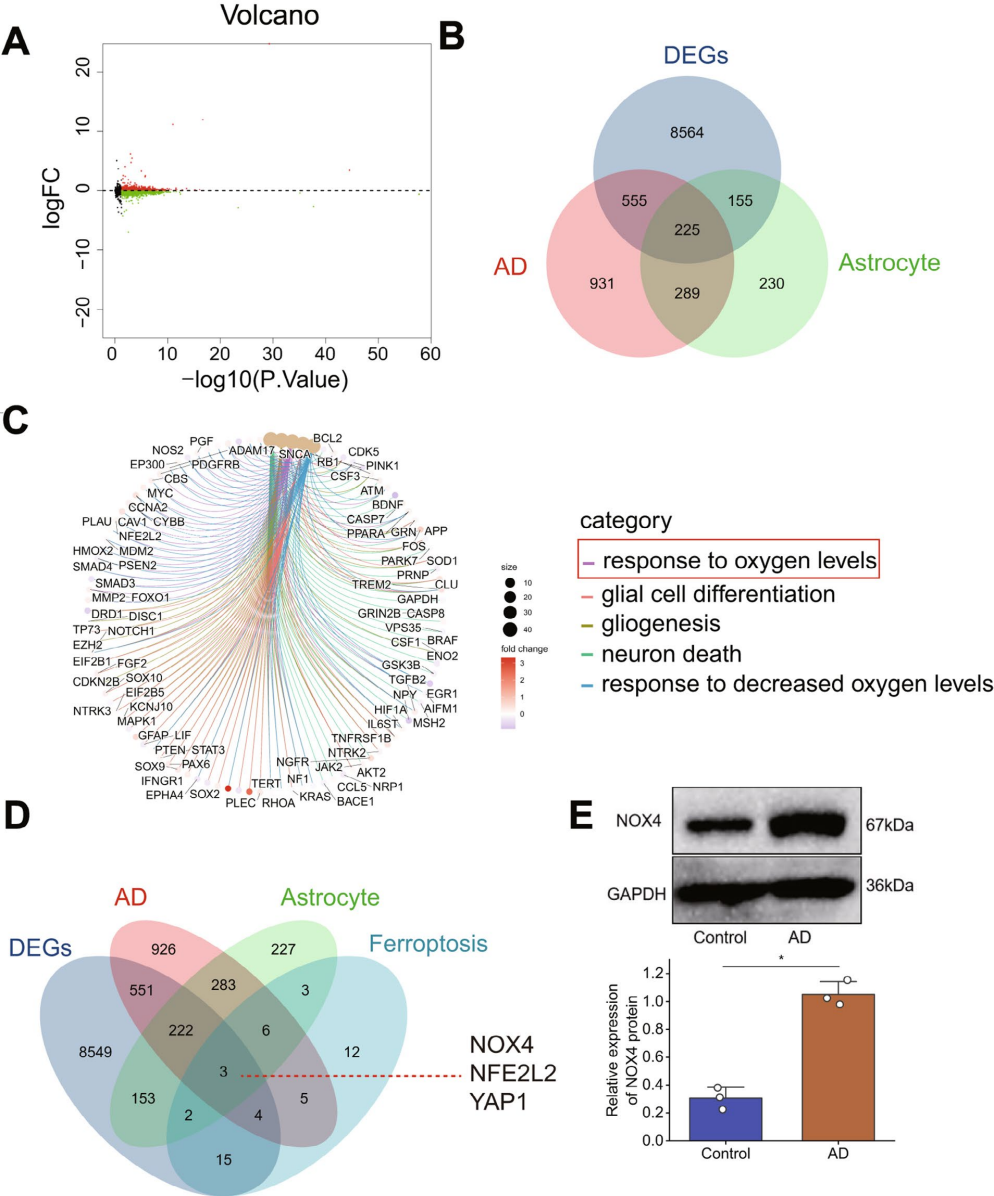
Identification of Target Genes in Alzheimer’s Disease Note: (**A**) Volcano plot of differentially expressed genes in transcriptome sequencing data (x-axis represents -log10 *p*-value, y-axis represents log FC, green dots represent downregulated genes, red dots represent upregulated genes, black dots represent no significant difference, Control group, *n* = 173; Model group, *n* = 80). (**B**) Venn diagram showing the intersection of DEGs and Genecards database-related genes in Alzheimer’s Disease (AD) and astrocytes. (**C**) GO enrichment analysis of AD-related differentially expressed genes in astrocytes (circle size indicates the number of enriched genes in the term, color represents enrichment *p*-value). (**D**) Venn diagram showing the intersection of DEGs and Genecards database-related genes in AD, astrocytes, and ferroptosis-related proteins. (**E**) WB detection of NOX4 expression levels in astrocytes of control and AD groups. **P < 0.01, all cellular experiments were repeated 3 times

To investigate the potential molecular mechanisms underlying the occurrence of Alzheimer’s disease, we screened the Alzheimer’s disease-related chip GSE48350 from the GEO database and obtained differentially expressed genes (DEGs) (Fig. 2A). We took the intersection of these DEGs with the top 2000 genes ranked by AD correlation score and the top 1000 genes ranked by astrocyte correlation score in the gene cards database (Fig. 2B) to obtain differentially expressed genes related to AD astrocytes. The GO enrichment analysis revealed (Fig. 2C) that these intersecting genes are enriched in pathways such as oxidative stress, neuronal death, and response to hypoxia.

Therefore, we intersected those mentioned above differentially expressed genes related to AD astrocytes with the top 50 ranking ferroptosis-related proteins in the Genecards database (Fig. 2D), resulting in three essential genes: NOX4, NFE2L2, and YAP1. NOX is considered to be the primary source of reactive oxygen species (ROS) production [67]. NOX4 is one of the main subtypes expressed in the central nervous system [68]. It is a crucial participant in the progression of multiple neurological disorders [28, 69, 70]. The expression level of NOX4 is increased in patients with Alzheimer’s disease and animal models. NOX4 promotes astrocyte ferroptosis through oxidative stress-induced lipid peroxidation [38, 71].

Further research and exploration into the role of NOX4 may help uncover the pathogenesis of Alzheimer’s disease and provide potential therapeutic strategies. We chose NOX4 as the target gene for subsequent experiments. We constructed an in vitro AD model by inducing normal human astrocytes with Aβ25–35 peptide. The WB results showed that, compared to the control group, the expression of NOX4 was significantly increased in the AD group (Fig. 2E). The above results indicate that the NOX4 gene may play an essential role in the occurrence and development of Alzheimer’s disease (AD).

### Elevated levels of NOX4 occur in astrocytes of 3.3 APP/PS1 mice, leading to ferroptosis

**Fig. 3:**
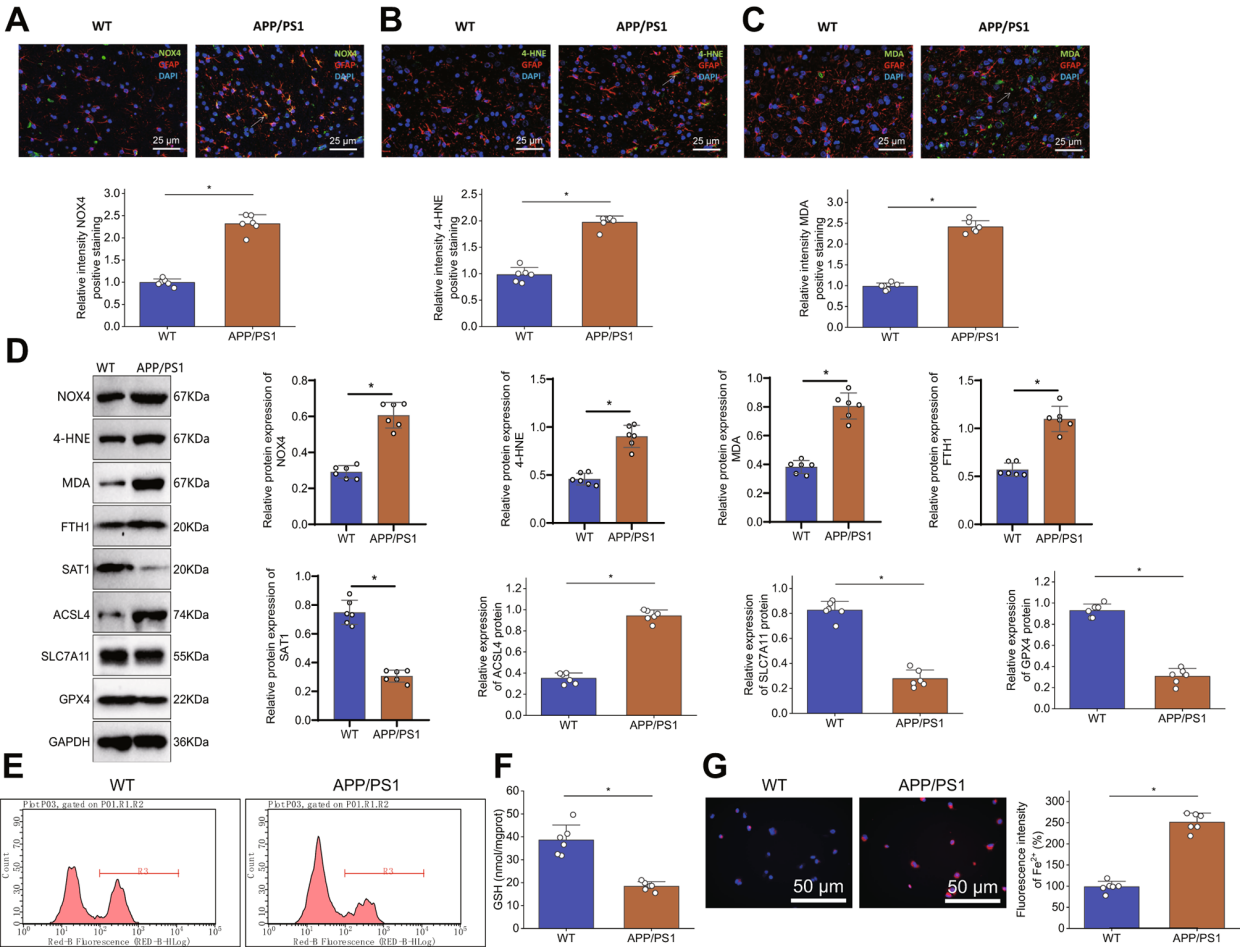
NOX4 and Ferroptosis in Astrocytes of APP/PS1 Mice Note: (**A**) Representative immunofluorescence images and statistical analysis of NOX4 expression in the cortical region of mice (astrocytes stained with GFAP in red, NOX4 positive staining in green, cell nuclei stained with DAPI in blue. Scale bar: 20 μm. White arrows indicate NOX4 and GFAP positive cells). (**B**-**C**) Representative immunofluorescence images and statistical analysis of 4-HNE and MDA in the cortical region of mice (astrocytes stained with GFAP in red, 4-HNE and MDA positive staining in green, cell nuclei stained with DAPI in blue. Scale bar: 20 μm. White arrows indicate 4-HNE, MDA, and GFAP positive cells). (**D**) WB detection of NOX4, 4-HNE, MDA, FTH1, SAT1, GPX4, SLC7A11, and ACSL4 expression levels in astrocytes of different groups of mice. (**E**) Detection of lipid peroxidation using C11-BODIPY 581/591 oxidative probe in astrocytes of different groups. (**F**) Measurement of GSH content in astrocytes of different groups. (**G**) Intracellular Fe2 + detection using FerroOrange (Scale bar: 25 μm). Each group contains 6 mice. ***P < 0.001

The relationship between NOX4 and iron death in astrocytes in Alzheimer’s disease (AD) is currently unclear. To investigate the role of NOX4 in Alzheimer’s disease, we conducted immunofluorescence staining to measure the levels of NOX4 protein in GFAP-positive astrocytes in the cortical region of APP/PS1 transgenic mice and wild-type (WT) mice. The results revealed an increase in the intensity of NOX4-positive staining in GFAP-positive astrocytes in the cortical region of APP/PS1 transgenic mice as shown in Fig. 3A.

Next, we analyzed whether the level of lipid peroxidation is elevated in the astrocytes of the cortical regions of APP/PS1 mice. Immunofluorescent staining results showed (Fig. 3B-C) that compared to wild-type mice, the intensity of 4-HNE and MDA-positive staining increased in GFAP-positive astrocytes in the cortical area of APP/PS1 mice. Subsequently, we used flow cytometry to isolate astrocytes from the brain cortex of mice, assessing the expression of NOX4, 4-HNE, and MDA. Additionally, we examined the status of cell iron death by measuring the expression of FTH1, SAT1, GPX4, SLC7A11, and ACSL4.

Western blotting results showed (Fig. 3D) that compared to wild-type mice, the expression levels of SAT1, GPX4 and SLC7A11 were decreased in the astrocytes of APP/PS1 mice, while the expression level of NOX4, 4-HNE, MDA, FTH1, and ACSL4 were increased. The results of lipid peroxidation detection showed (Fig. 3E) that, compared with the wild-type group, the degree of lipid peroxidation in astrocytes of the APP/PS1 group was enhanced. The GSH determination results showed (Fig. 3F) that the GSH content in APP/PS1 mice astrocytes decreased compared to wild-type mice. Iron ion content determination results showed (Fig. 3G) that compared with the wild-type group, the yellow fluorescence in star-shaped glial cells in the APP/PS1 group was enhanced, and the iron ion content was increased.

These results indicate that the levels of NOX4 in astrocytes of APP/PS1 mice are elevated, leading to ferroptosis.

### Silencing NOX4 could attenuate iron-induced astrocyte cell death

**Fig. 4:**
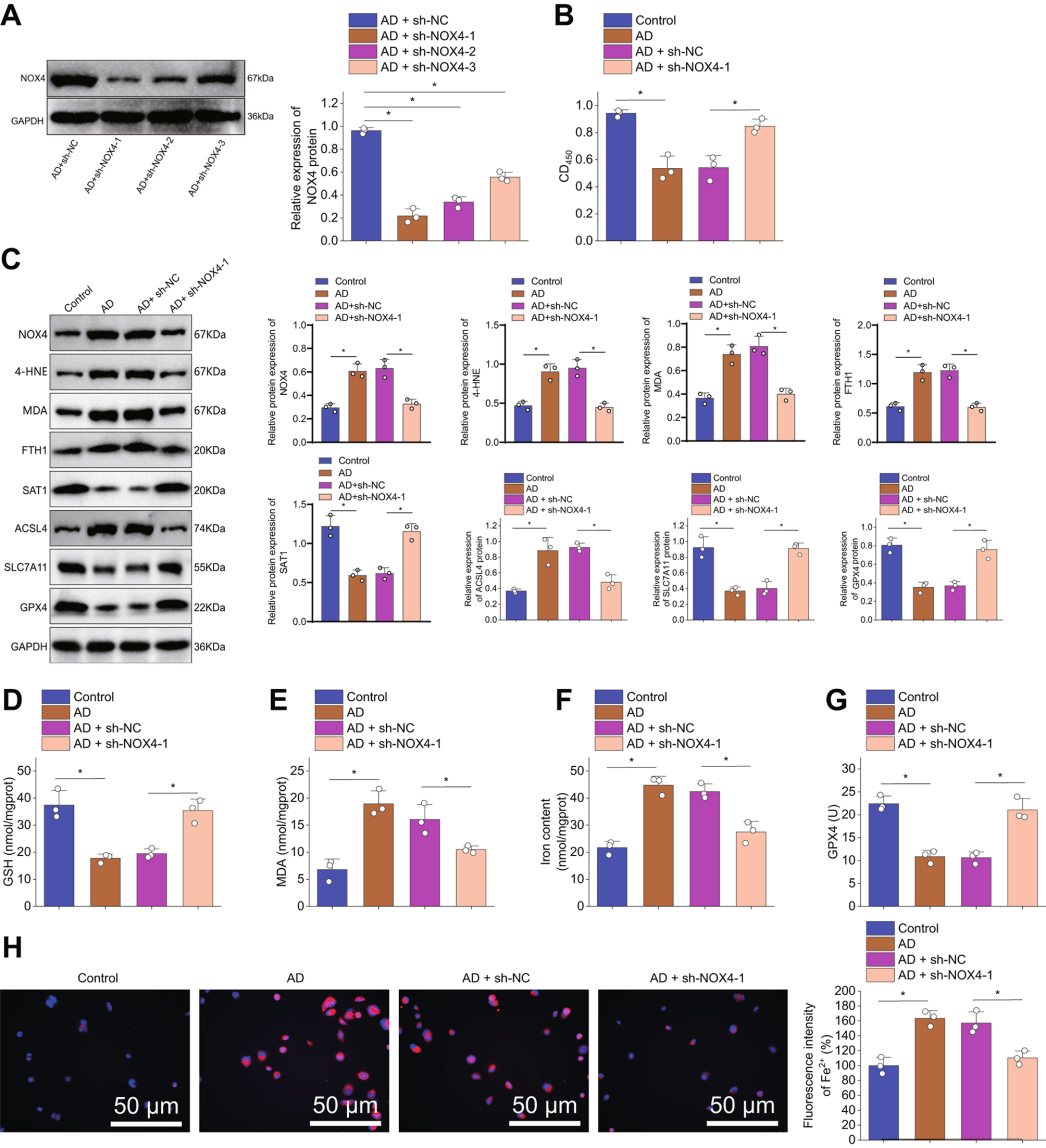
Effects of NOX4 Silencing on Ferroptosis in AD Cell Model Note: (**A**) Detection of NOX4 knockdown efficiency by WB after NOX4 silencing. (**B**) Cell viability of different groups of astrocytes detected using CCK-8 assay. (**C**) WB detection of NOX4, 4-HNE, MDA, FTH1, SAT1, GPX4, SLC7A11, and ACSL4 expression levels in astrocytes of different groups. (**D**) Measurement of GSH content in astrocytes of different groups. (**E**) Measurement of MDA content in astrocytes of different groups. (**F**) Measurement of iron ion content in astrocytes of different groups. (**G**) GPX4 enzyme activity in astrocytes of different groups. (**H**) Intracellular Fe2 + detection using FerroOrange (Scale bar: 25 μm). Note: *P* < 0.05, *P < 0.01, all cellular experiments were repeated 3 times

In the in vitro AD model experiments involving NOX4 silencing, the Western blot analysis results (Fig. 4A) indicated a significant reduction in NOX4 expression, with the most optimal silencing effect observed after the first attempt. The CCK-8 assay (Fig. 4B) showed a remarkable decrease in astrocyte activity in the AD model. Subsequent NOX4 silencing led to a significant enhancement in astrocyte activity. Additionally, we conducted Western blot detection of cell iron death-related proteins in this model. As shown in the results (Fig. 4C), the levels of SAT1, GPX4, and SLC7A11 in astrocytes of the AD model decreased, while the levels of NOX4, 4-HNE, MDA, FTH1, and ACSL4 increased. However, NOX4 silencing resulted in an increase in SAT1, GPX4, and SLC7A11 levels and a decrease in NOX4, 4-HNE, MDA, FTH1, and ACSL4 levels. GSH assay results (Fig. 4D) demonstrated a decrease in GSH content in astrocytes of the AD model, which significantly increased upon NOX4 silencing.

Analysis of MDA content (Fig. 4E) showed an increase in astrocytic MDA levels in the AD model, which significantly decreased upon NOX4 silencing. Iron ion content determination (Fig. 4F) revealed an increase in iron ion levels in astrocytes of the AD model, which significantly decreased following NOX4 silencing. Moreover, the GPX4 enzyme activity assay results (Fig. 4G) indicated a decrease in GPX4 enzyme activity levels in astrocytes of the AD model, which significantly increased upon NOX4 silencing. Finally, FerroOrange assay results (Fig. 4H) displayed enhanced yellow fluorescence and increased iron ion levels in astrocytes of the AD model, which decreased notably with NOX4 silencing, resulting in a significant reduction in iron ion content.

These findings suggest that NOX4 silencing can inhibit iron death in astrocytes.

### Silencing NOX4 could attenuate iron-induced cell death in APP/PS1 mice

To further investigate the impact of NOX on iron-induced cell death in APP/PS1 mice, we conducted a NOX4 knockdown experiment in the APP/PS1 mice.

**Fig. 5:**
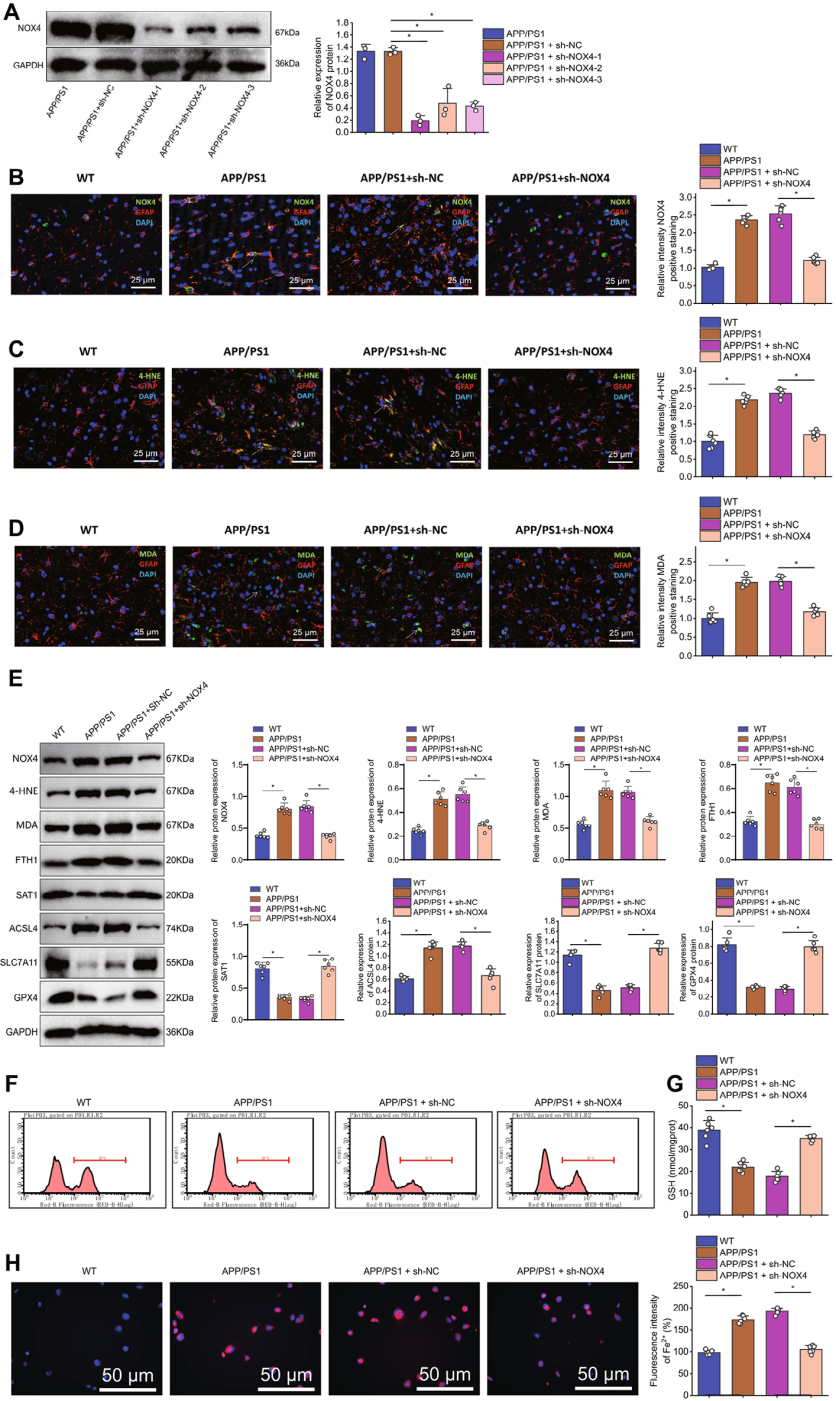
Effects of NOX4 Silencing on Ferroptosis in APP/PS1 Mice Note: (**A**) Detection of NOX4 knockdown efficiency by WB after NOX4 silencing. (**B**-**D**) Representative immunofluorescence images and statistical analysis of NOX4, 4-HNE, and MDA in the cortical region of mice (astrocytes stained with GFAP in red, NOX4, 4-HNE, and MDA positive staining in green, cell nuclei stained with DAPI in blue. Scale bar: 20 μm. White arrows indicate NOX4, 4-HNE, MDA, and GFAP-positive cells). (**E**) WB detection of NOX4, 4-HNE, MDA, FTH1, SAT1, GPX4, SLC7A11, and ACSL4 expression levels in astrocytes of different groups of mice. (**F**) Quantitative analysis of C11-BODIPY 581/591 oxidative probe fluorescence intensity using flow cytometry. (**G**) Measurement of GSH content in astrocytes of different groups. (**H**) Intracellular Fe2 + detection using FerroOrange (Scale bar: 25 μm). Each group contains 6 mice. ***P < 0.001

WB results revealed a significant decrease in NOX4 expression (Fig. 5A) after silencing NOX4. Among the groups, the first group exhibited the best silencing effect. Thus, we selected it for subsequent experiments. Immunofluorescent staining results showed that compared to the WT group, the intensity of NOX4, 4-HNE, and MDA-positive staining in GFAP-positive astrocytes in the cortical area increased in the APP/PS1 group (Fig. 5B-D). After silencing NOX4, the intensity of NOX4, 4-HNE, and MDA-positive staining decreased.

Later, we sorted mouse cortical astrocytes using flow cytometry and examined their ferroptosis status. The results of WB (Fig. 5E) showed that compared to the WT group, the expression levels of SAT1, GPX4 and SLC7A11 were decreased, while the expression level of NOX4, 4-HNE, MDA, FTH1 and ACSL4 was increased in astrocytes of the APP/PS1 group. After the silence of NOX4, the expression levels of SAT1, GPX4 and SLC7A11 increased, while the expression level of NOX4, 4-HNE, MDA, FTH1 and ACSL4 decreased. The results of lipid peroxidation detection showed that compared with the WT group (Fig. 5F), the degree of lipid peroxidation in astrocytes of the APP/PS1 group was enhanced. After silencing NOX4, the degree of lipid peroxidation decreased.

The GSH measurement results (Fig. 5G) showed that compared to the WT group, the GSH content in the astrocytes of the APP/PS1 group decreased. However, after silencing NOX4, the GSH content increased. The iron ion measurement results (Fig. 5H) showed that compared to the WT group, the yellow fluorescence in the astrocytes of the APP/PS1 group increased, indicating an increase in iron ion content. After silencing NOX4, the yellow fluorescence increased while the iron ion content decreased. These results suggest silencing NOX4 could inhibit ferroptosis in APP/PS1 mice.

### Silencing NOX4 improves APP/PS1 mouse models of Alzheimer’s disease

To investigate the potential of silencing NOX4 in mitigating iron-induced cell death and alleviating Alzheimer’s disease, we conducted experiments on APP/PS1 transgenic mice by employing NOX4 knockdown and erastin treatment. Initially, the Morris water maze test was carried out to assess spatial learning and memory in the mice. The findings revealed a prolonged escape latency in APP/PS1 transgenic mice compared to controls, whereas silencing NOX4 significantly reduced the escape latency in these mice.

Escape latency was prolonged in the APP/PS1 + sh-NOX4 + erastin group. In addition, the distances traveled by APP/PS1 mice in the target quadrant, the time spent within the target quadrant during the exploration test, and the number of passages through the platform were significantly reduced. However, these indicators were significantly improved after silencing NOX4.

**Fig. 6:**
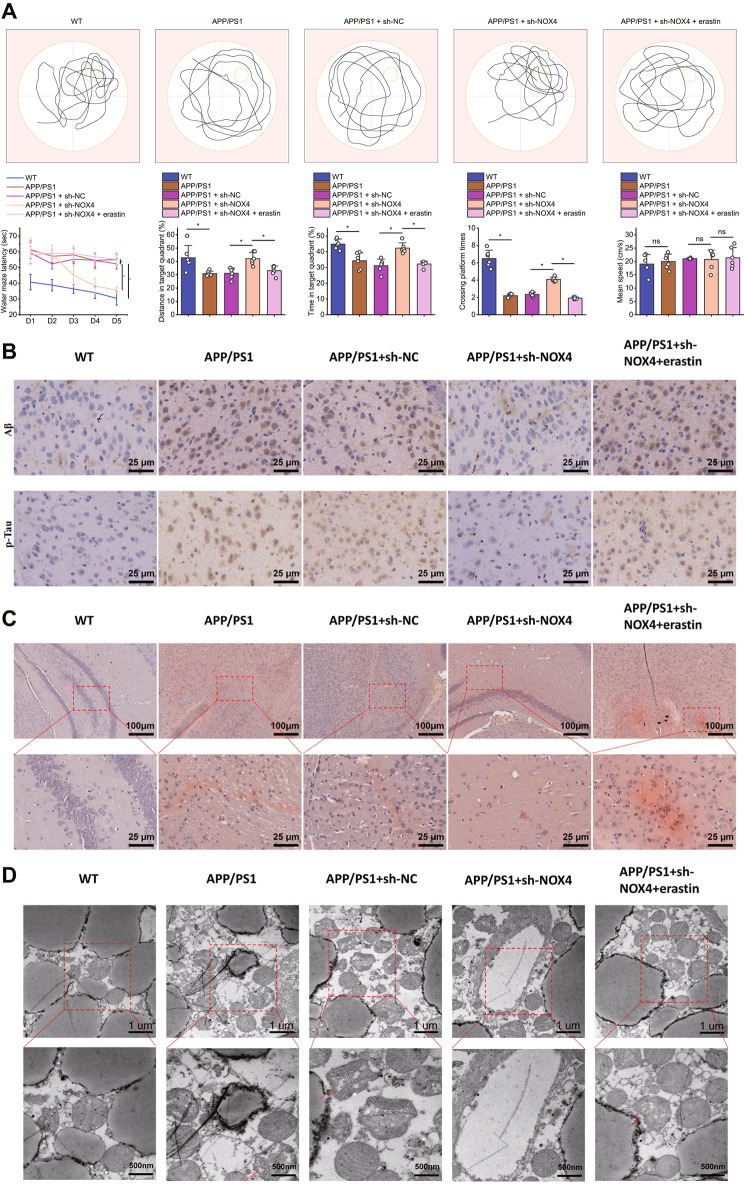
Effects of Silent NOX4 on Alzheimer’s Disease in APP/PS1 Mice Note: (**A**) Spatial learning and memory function of each group of mice were evaluated using the Morris water maze paradigm in this study. The figure presents the trajectories of mice in the Morris water maze, the escape latency of mice in the platform test on the fifth day, the percentage of distance traveled by mice to the target quadrant, the time spent by mice in the target quadrant, the number of platform crossings, and the swimming speed of mice in the Morris water maze. (**B**) The Aβ protein and p-Tau levels were detected using immunohistochemical methods in this study. The figures present the results with a scale of 100 μm. (**C**) This study detected the aggregation of amyloid plaques using Congo red staining. The figures present the results with scales of 500 μm and 100 μm. (**D**) The mitochondrial damage in the hippocampal region was observed using transmission electron microscopy (TEM) in this study. The arrows indicate mitochondrial damage. The figures present the results with scales of ×8000 and ×25,000. The study results show significant differences (*P* < 0.001) between the treatment groups and the control group in the mouse model. Each group consisted of 6 mice

There was a decrease in these indicators in the APP/PS1 + sh-NOX4 + erastin group. There was no significant difference in swimming speed among the groups of mice in the Morris water maze (Fig. 6A). Immunohistochemical analysis of Aβ protein and p-Tau levels revealed (Fig. 6B) significantly increased levels of Aβ and p-Tau proteins in the brains of APP/PS1 mice. However, silencing NOX4 significantly reduces the levels of Aβ and p-Tau proteins, while they are elevated in the APP/PS1 + sh-NOX4 + erastin group.

Congo red staining was used to detect the aggregation of amyloid protein plaques in the brains of APP/PS1 mice compared to WT mice (Fig. 6C). However, the aggregation of β-amyloid protein was reduced when NOX4 was silenced but increased in the APP/PS1 + sh-NOX4 + erastin group.

TEM results showed (Fig. 6D) that, compared to WT mice, the brains of APP/PS1 mice exhibited mitochondrial swelling and loss of mitochondrial cristae. However, the swelling of mitochondria and disappearance of mitochondrial cristae were reduced after silencing NOX4. However, in the APP/PS1 + sh-NOX4 + erastin group, mitochondrial swelling and mitochondrial cristae disappearance were more severe.

The above results indicate that silencing NOX4 could improve Alzheimer’s disease in APP/PS1 mice.

## Discussion

The results of this study offer novel insights into the pathogenesis of Alzheimer’s disease (AD). Our research successfully unveils the pivotal role of NOX4 in iron-induced astrocytic cell death, leveraging single-cell sequencing technology. This discovery is of paramount significance since, to our knowledge, although astrocytes have been recognized as key players in AD’s progression, their precise functions and the specific interplay between astrocytes and AD development remain largely obscure [72, 73]. Our findings have unveiled a potential relationship between NOX4 and iron-induced cell death in Alzheimer’s disease, offering a novel insight into unraveling the intricate pathogenesis of Alzheimer’s.

Prior investigations have extensively demonstrated the critical role of iron metabolism dysregulation and oxidative stress in AD. However, these studies have predominantly explored global phenotypes and overarching mechanisms, with relatively limited scrutiny of specific cell types and cellular states [23, 74, 75]. In our study, single-cell sequencing technology played a vital role [35, 76]. This approach enabled us to investigate the transcriptomic expression at a single-cell level, providing more detailed information compared to conventional whole-sample-based research methods [77]. Undoubtedly, this significantly enhanced the depth and breadth of our study. Our research unveiled, for the first time, the high expression of NOX4 in astrocytes in AD. Subsequent in vitro and in vivo experiments confirmed that elevated NOX4 expression in astrocytes led to iron-induced cell death.

Historically, the concept of iron-induced cell death has been predominantly applied in cancer research, with relatively limited exploration in neurodegenerative diseases, particularly Alzheimer’s disease [25, 78, 79]. Our study uncovered the significant role of iron-induced cell death in Alzheimer’s disease, establishing a connection between astrocytes and iron-induced cell death, thus forging a new path for investigating the relationship between iron-induced cell death and neurodegenerative diseases.

**Fig. 7:**
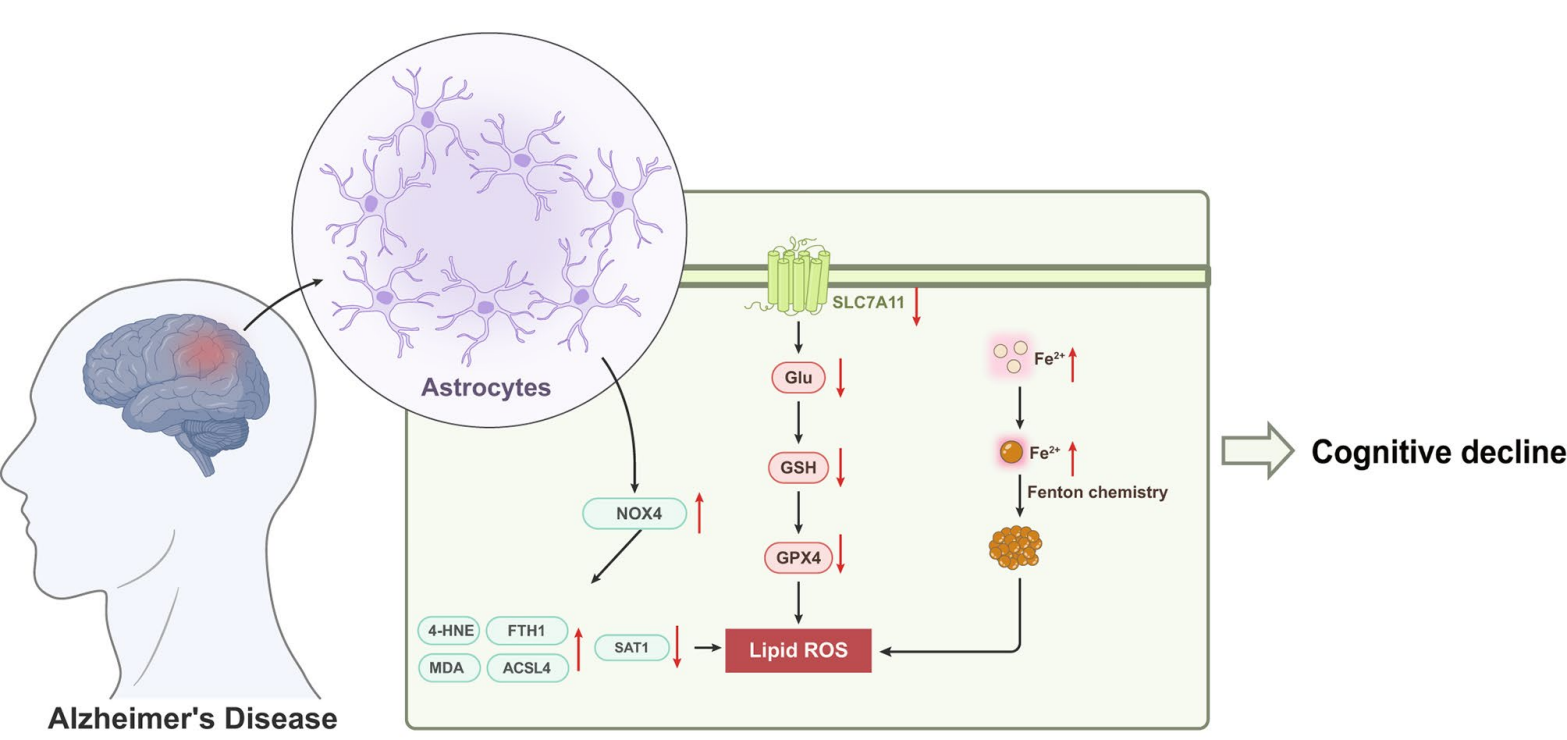
Molecular mechanism diagram of NOX4 mediated astrocyte ferroptosis promoting Alzheimer’s disease

Based on the aforementioned results, we tentatively conclude that NOX4 mediates astrocytic ferroptosis and fosters Alzheimer’s disease progression (Fig. 7). This study has revealed that NOX4 (NADPH oxidase 4) plays a crucial role in the iron-induced cell death process of astrocytes. Silencing NOX4 can effectively inhibit iron-induced cell death, consequently enhancing spatial learning and memory functions in AD mice while reducing levels of Aβ and p-Tau proteins. These findings offer a novel potential for the treatment of Alzheimer’s disease. From a clinical perspective, our discovery presents a promising therapeutic target.

One strength of this study lies in the precise identification of the differential gene NOX4 in Alzheimer’s disease patients using single-cell sequencing technology, linking astrocytes with iron-induced cell death and offering a new perspective on the complex pathogenesis of Alzheimer’s disease. While the study has yielded some positive results, there are still some limitations to address. Firstly, our reliance on publicly available databases for gene expression analysis and screening may introduce inherent biases [80]. Secondly, while we have elucidated NOX4’s role in iron-induced astrocytic cell death in AD, further research is essential to uncover its specific regulatory mechanisms. Moreover, our study predominantly relies on a mouse model for experimental validation, necessitating further confirmation in human studies. Future investigations should delve into the precise biological mechanisms through which NOX4 governs ferroptosis in astrocytes. Previous studies have indicated that TNFα acts as an upstream factor of NOX4, regulating the expression of NOX4 [81–84]. Therefore, further research is needed to determine whether NOX4 is the most direct target. Subsequent experiments could investigate the impact of TNFα regulation on NOX4 in astrocytes, exploring its influence on iron-induced cell death and its role in Alzheimer’s disease. Additionally, comprehensive and extensive clinical studies are warranted to corroborate our findings and ascertain whether NOX4 represents a viable therapeutic target. Furthermore, the potential of single-cell sequencing technology to unravel the microscopic underpinnings of Alzheimer’s disease progression beckons further exploration. In conclusion, our research paves new pathways and offers hope for a deeper comprehension of Alzheimer’s disease pathogenesis and the development of innovative AD treatments.

### Electronic supplementary material

Below is the link to the electronic supplementary material.

Supplementary Material 1

Supplementary Material 2

Supplementary Material 3

## Data Availability

The data that supports the findings of this study are available on request from the corresponding author.
