# Supplementary material for: NOX4-mediated astrocyte ferroptosis in Alzheimer’s disease
Source: Cell Biosci. 2024 Jul 2;14:88. doi: 10.1186/s13578-024-01266-w (PMC11218381; doi:10.1186/s13578-024-01266-w)
Supplement: Supplementary file 1 — Supplementary Material 1 [file 13578_2024_1266_MOESM1_ESM.docx]

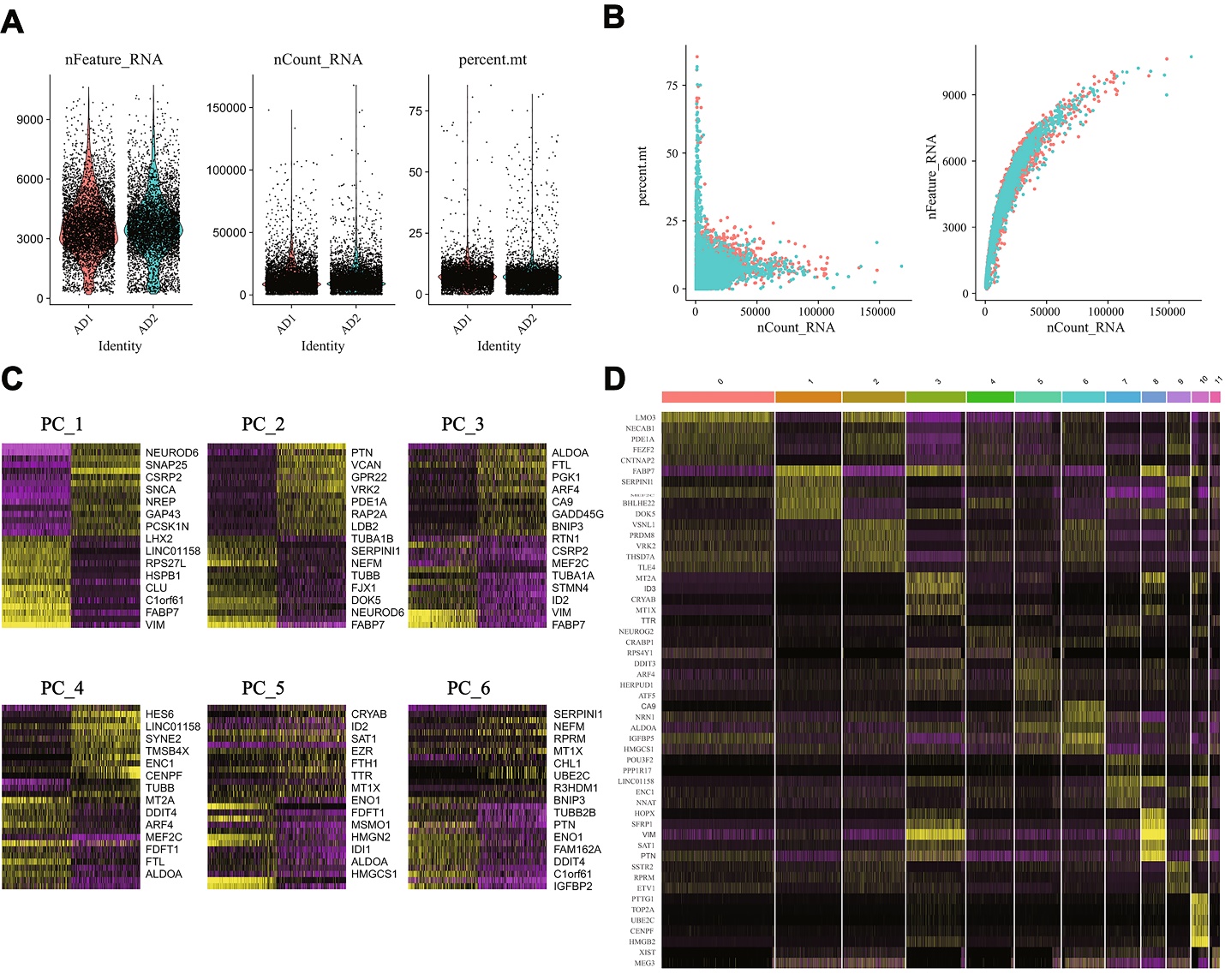


**Figure S1. Quality Control and PCA Dimensionality Reduction of scRNA-seq Data.**

Note: (A) Violin plots of scRNA-seq data show the number of genes (nFeature_RNA), number of mRNA molecules (nCount_RNA), and percentage of mitochondrial genes (percent. mt) in each cell. (B) Filtered data (nCount_RNA and percent. mt), as well as the scatter plot showing the correlation between nCount_RNA and nFeature_RNA (red represents sample GSM4996461 (AD1), blue represents sample GSM4996463 (AD2)). (C) Heatmap of the top 20 most correlated gene expressions in PC_1 - PC_6 in the PCA, where yellow indicates upregulation and purple indicates downregulation. (D) Heatmap of the top 5 marker gene expressions in each cluster, where yellow indicates upregulation and purple indicates downregulation.
