# Supplementary material for: NOX4-mediated astrocyte ferroptosis in Alzheimer’s disease
Source: Cell Biosci. 2024 Jul 2;14:88. doi: 10.1186/s13578-024-01266-w (PMC11218381; doi:10.1186/s13578-024-01266-w)
Supplement: Supplementary file 2 — Supplementary Material 2 [file 13578_2024_1266_MOESM2_ESM.doc]

**Table S1 shRNAsequence**

| Name | shRNA sequence |
| --- | --- |
| shRNA-NC | Sense 5’-UAAGGCUAUGAAGAGAUAC-3’ |
|  | Antisense 5’-GUAUCUCUUCAUAGCCUUA-3’ |
| shRNA-NOX4-1(human) | Sense 5’-GCTGAAGTATCAAACTAATTT-3’ |
|  | Antisense 5’-AAATTAGTTTGATACTTCAGC-3’ |
| shRNA-NOX4-2(human) | Sense 5’-CGAACCAGCTCTCAGAATATT-3’ |
|  | Antisense 5’-AATATTCTGAGAGCTGGTTCG-3’ |
| shRNA-NOX4-3(human) | Sense 5’-GAACCAGCTCTCAGAATATTT-3’ |
|  | Antisense 5’-AAATATTCTGAGAGCTGGTTC-3’ |
| shRNA-NOX4-1(mouse) | Sense 5’-CGCAATAAGAGTTTCTAATTA-3’ |
|  | Antisense 5’-TAATTAGAAACTCTTATTGCG-3’ |
| shRNA-NOX4-2(mouse) | Sense 5’-CTGGACCTTTGTGCCTTTATT-3’ |
|  | Antisense 5’-AATAAAGGCACAAAGGTCCAG-3’ |
| shRNA-NOX4-3(mouse) | Sense 5’-GCAAGACCTGGTCAGTATATT-3’ |
|  | Antisense 5’-AATATACTGACCAGGTCTTGC-3’ |
